# Desferrioxamine reduces ultrahigh-molecular-weight polyethylene-induced osteolysis by restraining inflammatory osteoclastogenesis via heme oxygenase-1

**DOI:** 10.1038/cddis.2016.339

**Published:** 2016-10-27

**Authors:** Hui Kang, Yufei Yan, Peng Jia, Kai Yang, Changjun Guo, Hao Chen, Jin Qi, Niandong Qian, Xing Xu, Fei Wang, Changwei Li, Lei Guo, Lianfu Deng

**Affiliations:** 1 Shanghai Key Laboratory for Bone and Joint Diseases, Shanghai Institute of Orthopaedics and Traumatology, Shanghai Ruijin Hospital, Shanghai Jiaotong University School of Medicine, Shanghai, China; 2 Department of Orthopaedics, The Second Affiliated Hospital of Soochow University, Suzhou, Jiangsu Province, China

## Abstract

As wear particles-induced osteolysis still remains the leading cause of early implant loosening in endoprosthetic surgery, and promotion of osteoclastogenesis by wear particles has been confirmed to be responsible for osteolysis. Therapeutic agents targeting osteoclasts formation are considered for the treatment of wear particles-induced osteolysis. In the present study, we demonstrated for the first time that desferrioxamine (DFO), a powerful iron chelator, could significantly alleviate osteolysis in an ultrahigh-molecular-weight polyethylene (UHMWPE) particles-induced mice calvaria osteolysis model. Furthermore, DFO attenuated calvaria osteolysis by restraining enhanced inflammatory osteoclastogenesis induced by UHMWPE particles. Consistent with the *in vivo* results, we found DFO was also able to inhibit osteoclastogenesis in a dose-dependent manner *in vitro*, as evidenced by reduction of osteoclasts formation and suppression of osteoclast specific genes expression. In addition, DFO dampened osteoclasts differentiation and formation at early stage but not at late stage. Mechanistically, the reduction of osteoclastogenesis by DFO was due to increased heme oxygenase-1 (HO-1) expression, as decreased osteoclasts formation induced by DFO was significantly restored after *HO-1* was silenced by siRNA, while HO-1 agonist COPP treatment enhanced DFO-induced osteoclastogenesis inhibition. In addition, blocking of p38 mitogen-activated protein kinase (p38MAPK) signaling pathway promoted DFO-induced HO-1 expression, implicating that p38 signaling pathway was involved in DFO-mediated HO-1 expression. Taken together, our results suggested that DFO inhibited UHMWPE particles-induced osteolysis by restraining inflammatory osteoclastogenesis through upregulation of HO-1 via p38MAPK pathway. Thus, DFO might be used as an innovative and safe therapeutic alternative for treating wear particles-induced aseptic loosening.

Artificial joint replacement has emerged as an effective treatment for severe joint degeneration.^1^ Although much effort has been made to improve the efficacy of artificial joint replacement, ultrahigh-molecular-weight polyethylene (UHMWPE) wear particles-induced osteolysis still remains the leading cause of early implant loosening in endoprosthetic surgery.^2, 3, 4^ Although the underlying mechanisms by which UHMWPE wear particles promoted-osteolysis are not fully elucidated, studies have showed that osteolysis at the periprosthetic site is dominantly due to the enhanced osteoclastic resorption activity.^5, 6^

Normal bone remodeling maintains constant bone mass by an orchestrated balance between the destruction of old bone by osteoclasts and rebuilding by osteoblasts.^7^ Osteoclasts, arising from hematopoietic stem cells, are the sole bone-resorbing cells.^8, 9, 10^ Osteoclasts undergo differentiation and fusion resulting in large multinucleated cells in the presence of receptor activator of nuclear factor-*κ*B ligand (RANKL) and macrophage-colony stimulating factor (M-CSF).^11^ Wear particles can stimulate macrophages, phagocytes and T lymphocytes to produce high concentrations of chemokines and cytokines, such as M-CSF, interleukin (IL)-1, IL-6, prostaglandin E2 (PGE2) and tumor necrosis factor-*α* (TNF-*α*), which lead to increase of RANKL and/or have direct effects on osteoclastogenesis and bone resorption.^12, 13, 14^ RANKL binds to its receptor RANK, resulting in a cascade of intracellular events, such as the activation of nuclear factor-*κ*B (NF-*κ*B) signaling pathway, the mitogen-activated protein kinases (MAPKs) signaling pathways and the nuclear factor of activated T-cells1 (NFATc1) signaling pathway, which are essential for osteoclast formation.^15, 16, 17^ Furthermore, wear particles also result in the production of reactive oxygen species (ROS), which induces oxidative stress and has a major role in regulating osteoclast function and bone resorption.^18^ Heme oxygenase-1 (HO-1), the rate-limiting step in heme catabolism, besides of working as a negative regulator of inflammation and oxidative stress, which has also been demonstrated as an osteoclastogenesis suppressor.^19^ Furthermore, HO-1 dampens early differentiation of osteoclast precursors into osteoclasts, but not acts on mature osteoclasts.^19, 20^ In addition to the cytokines and growth factors, it is reported that iron homeostasis may contribute to fine-turning of the RANKL-induced osteoclast development.^21, 22^ In general, inhibitions of osteoclasts formation and/or function by modulating microenvironmental cytokines, growth factors, HO-1 and/or iron homeostasis may be critical for preventing from wear particles-induced osteolysis and pathological bone loss.

Desferrioxamine (DFO) is a trihidroxamate, natural siderophore, capable of chelating iron, aluminum and other trivalent metallic ions forming stable chemical complexes.^23^ DFO has been widely used as a therapeutic agent for treating iron overload diseases.^24^ Growing evidences suggest that DFO can regulate the osteoblasts proliferation and differentiation and inhibit the osteoclasts formation.^25, 26, 27^ Therefore, DFO may be used as a therapeutic agent for the treatment of bone metabolic disease, such as osteoporosis. However, it remains unclear whether DFO can prevent UHMWPE particles-induced osteolytic diseases *in vivo*.

In the present study, we demonstrated for the first time that DFO could significantly alleviate particles-induced osteolysis in an UHMWPE particles-induced mouse calvaria osteolysis model. Furthermore, UHMWPE wear particles-induced osteoclastogenesis in the eroded bone surface was significantly attenuated by the treatment of DFO, which suggested DFO prevented UHMWPE particles-induced osteolysis by inhibition of osteoclast function and formation. Subsequently, we accomplished a series of biochemical and morphological studies to explore the effect of DFO on osteoclastogenesis. We found that DFO was able to inhibit osteoclastogenesis in a dose-dependent manner. Mechanistically, the reduction of osteoclastogenesis by DFO was due to increasing of HO-1 expression. In addition, blocking of p38 signaling pathway promoted DFO-induced HO-1 expression, implicating that p38 signaling pathway was involved in DFO-mediated HO-1 expression. Taken together, our results suggested that DFO could potentially be served as an alternative therapeutic option for UHMWPE particles-induced osteolysis.

## Results

### DFO alleviated UHMWPE particles-induced osteolysis *in vivo*

**Figure 1:**
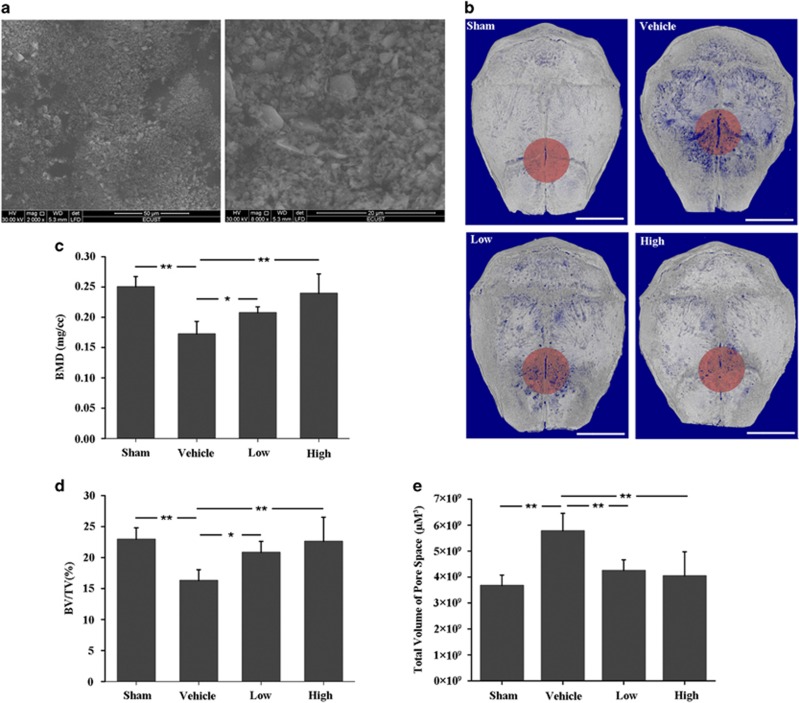
DFO alleviated UHMWPE particles-induced mouse calvaria osteolysis. (**a**) Scanning electron micrograph of UHMWPE particles. (**b**) Representative micro-CT three-dimensional reconstructed images from each group. Scale bars, 3 mm. (**c**–**e**) BMD, BV/TV and total volume of pore space in the region of interest were measured. Low and high represent 10 and 30 mg/kg DFO application, respectively. *n*=6, *P<0.05, **P<0.01

A murine calvaria osteolysis model was used to observe the effect of DFO on UHMWPE particles (Figure 1a)-induced osteolysis. Micro-CT analysis showed that extensive bone resorption was presented in the UHMWPE particles group (Vehicle), which was significantly attenuated by DFO treatment in a dose-dependent manner (Figure 1b). Furthermore, BMD, BV/TV and total volume of pore space in the region of interest (ROI) were also measured. The results showed that osteolysis was significantly increased in the vehicle group compared with sham control, while DFO injection with 10 mg/kg (low) or 30 mg/kg (high) daily could significantly prevent from UHMWPE particles-induced osteolysis (Figures 1c–e).

**Figure 2:**
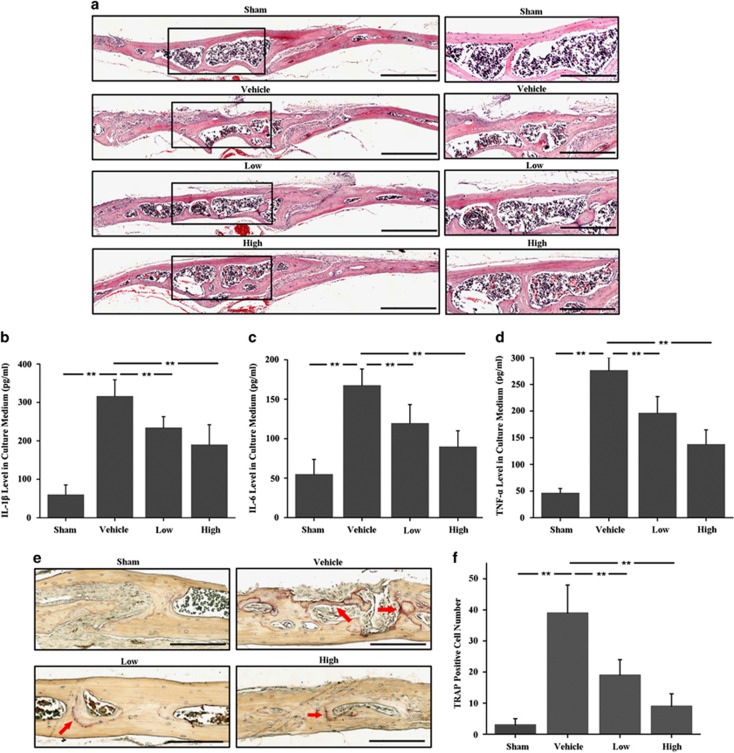
The inhibitory effects of DFO on UHMWPE particles-induced mouse calvarial osteolysis were observed by histological and histomorphometric analysis. (**a**) H&E staining showed much more inflammatory reaction and prominent osteolysis in vehicle group compared with sham group, while the DFO-treated groups exhibited reduced inflammation and osteolysis. Scale bars, 500 *μ*m. The rightmost pictures designate the larger magnification of the regions shown in inset. Scale bars, 300 *μ*m. (**b**–**d**) The concentration of IL-1*β* (Figure 2b), IL-6 (Figure 2c) and TNF-*α* (Figure 2d) in the supernatant after 72 h of calvaria culture detected by ELISA. (**e**,**f**) TRAP staining showed that the number of osteoclasts lined along the eroded bone surface was significantly increased in UHMWPE particles group, which was obviously reduced in both low and high concentrations of DFO-treated groups. Red arrows indicated TRAP-positive cells. Low and high represent 10 and 30 mg/kg DFO application, respectively. Scale bars, 300 *μ*m. *n*=6, **P<0.01

Subsequently, histological assessment and histomorphometric analysis were accomplished to detect the effect of DFO on UHMWPE particles-induced osteolysis. Hematoxylin and eosin (H&E) staining showed that there were much more inflammatory responses and prominent osteolysis in vehicle group compared with sham group, while the DFO-treated groups exhibited reduced inflammatory responses and osteolysis (Figure 2a). Consistent with the histological results, the calvarias culture results also confirmed that DFO significantly dampened particles-induced inflammatory responses, as the increased IL-1*β* (Figure 2b), IL-6 (Figure 2c) and TNF-*α* (Figure 2d) expression in the particles group were all abundantly decreased after DFO treatment. Furthermore, TRAP staining showed that the number of osteoclasts lined along the eroded bone surface was significantly increased in vehicle group compared with sham group, but which was obviously reduced in both low (10 mg/kg) and high (30 mg/kg) concentrations of DFO-treated groups (Figures 2e and f). Taken together, these results suggested that DFO treatment could markedly protect from UHMWPE particles-induced osteolysis via dampening inflammatory osteoclastogenesis *in vivo.*

### DFO inhibited osteoclastogenesis *in vitro*

**Figure 3:**
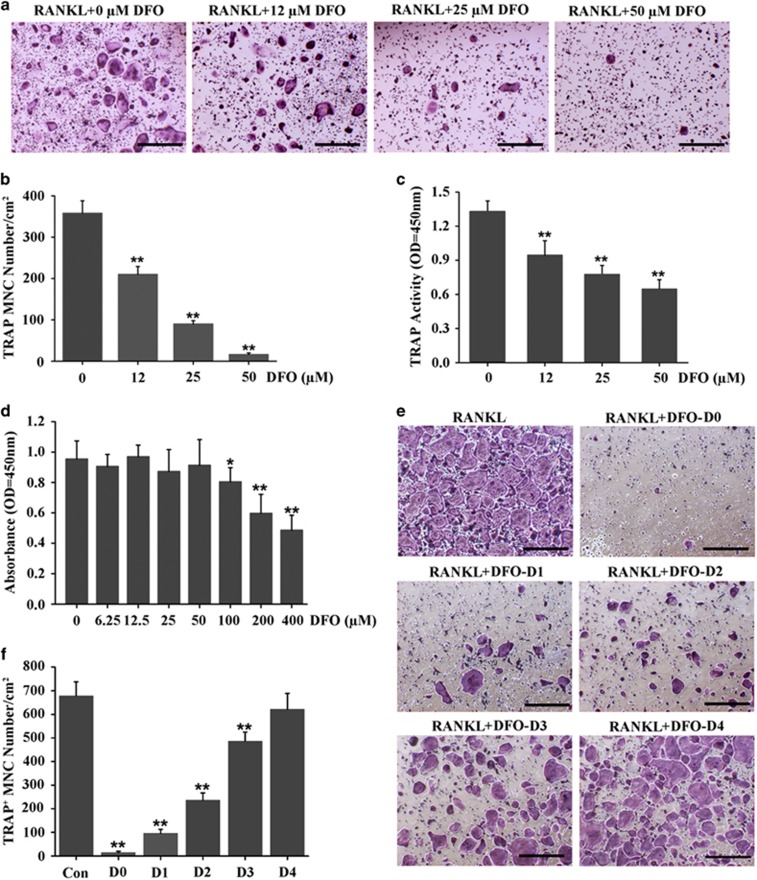
DFO inhibited osteoclastogenesis *in vitro.* (**a**) BMMs were induced with 30 ng/ml M-CSF and 50 ng/ml RANKL in the presence of different concentrations of DFO for 5 days, followed by staining with TRAP. Scale bars, 50 *μ*m. (**b**) The number and area of TRAP-positive cells was counted. *n*=4, **P<0.01. (**c**) TRAP activity assessment was accomplished in BMMs treated with different concentrations of DFO during osteoclastogenesis. *n*=4, **P<0.01. (**d**) CCK-8 assay was performed to examine the effect of different concentrations of DFO on cells viability. *n*=6, *P<0.05, **P<0.01. (**e**) TRAP staining was performed in BMMs treated with DFO at different stage during osteoclastogenesis. Scale bars, 50 *μ*m. (**f**) The number and area of TRAP-positive cells was counted. *n*=4, **P<0.01

Having observed DFO attenuated UHMWPE particles-induced osteolysis by suppression of osteoclastogenesis *in vivo*, we next detected the effect of DFO on osteoclasts formation *in vitro*. Bone marrow-derived macrophages (BMMs) were induced with 30 ng/ml M-CSF and 50 ng/ml RANKL in the presence of different concentrations of DFO for 5 days. The results of TRAP staining showed that the number of mature osteoclasts was significantly decreased by DFO in a dose-dependent manner. Approximately 50% fewer of TRAP-positive osteoclasts were observed in cells treated with 12 *μ*M DFO compared with the control group, and there were almost no mature osteoclasts after 50*μ*M DFO treatment (Figures 3a and b). Consistent with the results of TRAP staining, DFO also inhibited TRAP activity of osteoclasts in a dose-dependent manner (Figure 3c). To determine whether DFO-inhibited osteoclastogenesis was due to the cytotoxic effects of DFO, we performed a CCK-8 assay to examine the effect of DFO on cells viability. The results showed that no significant cytotoxic effect was observed in BMMs treated with DFO, even at concentration up to 50 *μ*M (Figure 3d), suggesting that DFO could inhibit osteoclasts formation without any cytotoxic effects. To further examine at which stage DFO inhibited osteoclastogenesis, 50 *μ*M DFO was added into culture medium at 0–4 days during osteoclastogenesis. The results showed that DFO could significantly inhibit osteoclasts formation at early stage (days 0–3), whereas adding DFO to osteoclastic precursor cells at late stage (day 4) could not affect osteoclasts formation, which predicted that DFO inhibited osteoclasts differentiation at early stage but not at late stage (Figures 3e and f).

**Figure 4:**
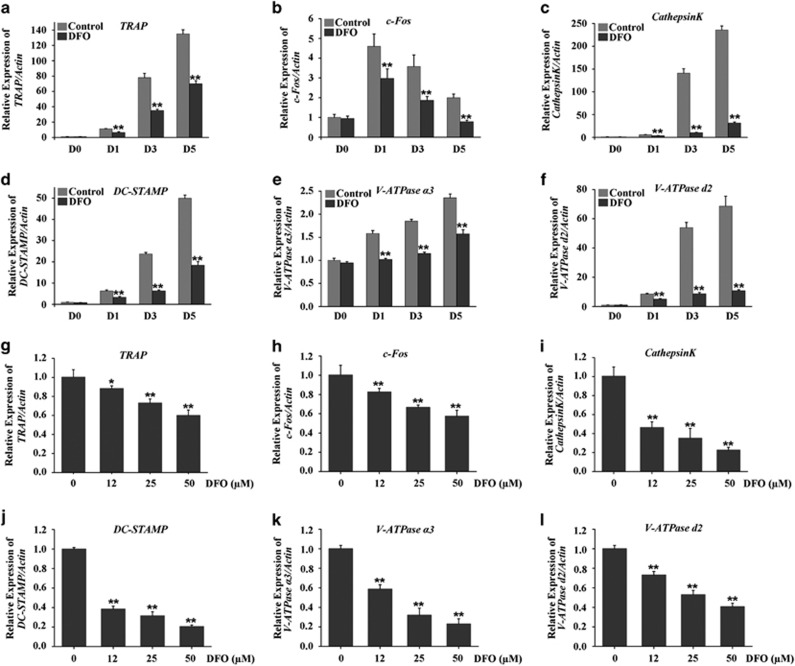
DFO inhibits RANKL-induced osteoclast-specific gene expression. (**a**–**f**) qRT-PCR analysis of osteoclasts formation specific genes, *TRAP*, *c-Fos*, *Cathepsin K*, *DC-STAMP*, *V-ATPase a3* and *V-ATPase d2*, in BMMs treated with 50 *μ*M DFO at different stage during osteoclastogenesis. *n*=4, **P<0.01. (**g**–**l**) qRT-PCR analysis of osteoclasts formation specific genes, *TRAP*, *c-Fos*, *Cathepsin K*, *DC-STAMP*, *V-ATPase a3* and *V-ATPase d2*, in BMMs treated with different concentrations of DFO during osteoclastogenesis. *n*=4, **P<0.01

A set of genes have been found to be associated with osteoclasts differentiation and formation, such as *TRAP*, *c-Fos*, *Cathepsin K*, *DC-STAMP*, *V-ATPase a3* and *V-ATPase d2.*^28^ Therefore, to further examine the inhibitory effect of DFO on osteoclasts formation, we detected the effects of DFO on these genes expression. Our results showed that these genes expression were obviously upregulated during RANKL-induced osteoclasts formation, whereas which were all markedly suppressed by 50 *μ*M DFO in a time-dependent manner (Figures 4a–f). Furthermore, DFO also inhibited these genes expression in a dose-dependent manner (Figures 4g–l). Taken together, these results further strengthened our conclusion that DFO could decrease osteoclasts formation.

### DFO inhibited osteoclastic bone resorption and F-actin ring formation

**Figure 5:**
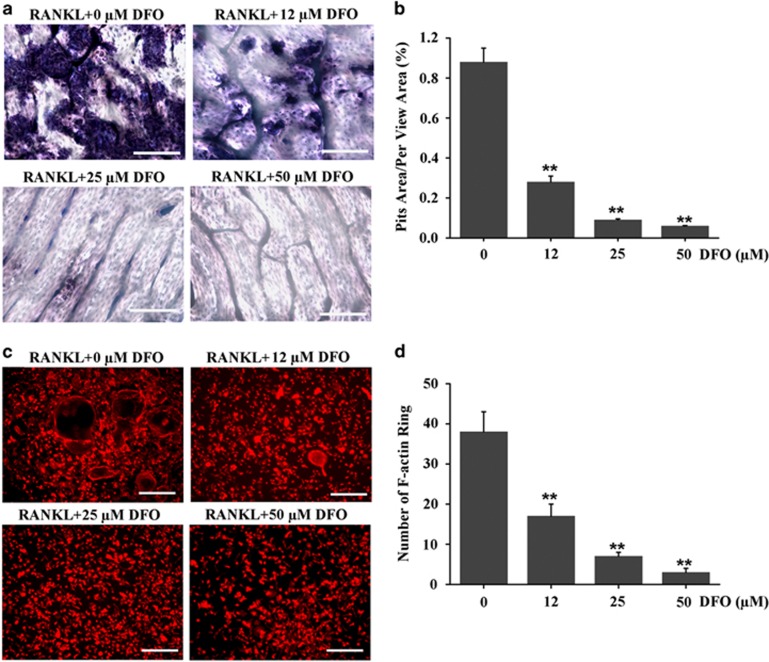
DFO inhibited osteoclastic bone resorption and F-actin ring formation. (**a**) Resorption pit formation in BMMs treated with different concentrations of DFO during osteoclastogenesis. Scale bars, 20 *μ*m. (**b**) Summarized data showed that DFO significantly decreased osteoclasts bone resorption in a dose-dependent manner. *n*=4, **P<0.01. (**c**) F-actin ring staining was performed to estimate the effect of different concentrations of DFO on osteoclastic bone resorption. Scale bars, 50 *μ*m. (**d**) Summarized data showed DFO significantly decreased number of F-actin ring in a dose-dependent manner. *n*=4, **P<0.01

Even though DFO could impair osteoclasts formation, it was unclear whether DFO could inhibit osteoclasts activity. Therefore, we performed pit formation assay to estimate the effect of DFO on osteoclastic bone resorption. BMMs were cultured on bone slices, and induced by M-CSF and RANKL in the presence of different concentrations of DFO for 10 days. We found a significant increase of pits formation in the control group. However, the resorption area was markedly decreased in DFO-treatment group. Furthermore, we found DFO inhibited osteoclastic bone resorption in a dose-dependent manner, as the resorption area decreased by 60% after 12 *μ*M DFO treatment, and there was almost no obvious pits formation when the concentration of DFO reached to 25 *μ*M DFO and 50 *μ*M (Figures 5a and b). In addition, a well-polarized F-actin ring was required for efficient bone resorption. Therefore, we performed F-actin ring staining to estimate the effect of DFO on osteoclastic bone resorption. The clear F-actin ring structures were observed in the untreated control group (Figures 5c and d). However, the F-actin ring structures were significantly disrupted when BMMs incubated with 12, 25 or 50 *μ*M DFO (Figures 5c and d). Taken together, all these results demonstrated that DFO could inhibit osteoclastic bone resorption.

### DFO mediated osteoclastogenesis by regulating HO-1 expression

**Figure 6:**
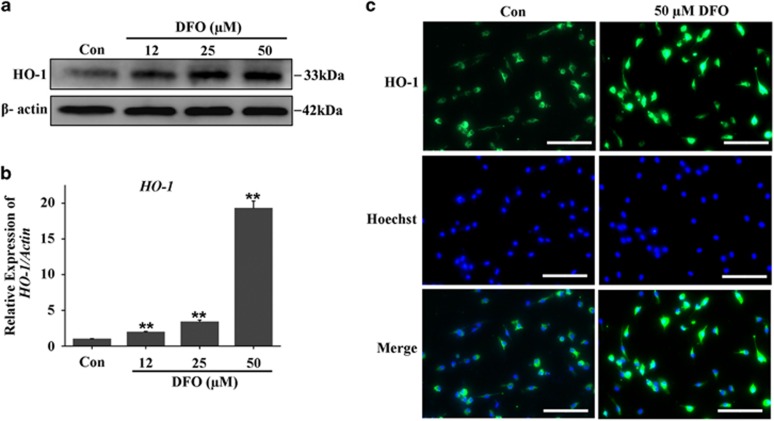
DFO-induced HO-1 expression during osteoclastogenesis. (**a**) Western blot analysis showed that DFO could induce HO-1 protein expression in a dose-dependent manner. (**b**) qRT-PCR analysis showed that DFO could induce HO-1 *mRNA* expression in a dose-dependent manner. *n*=4, **P<0.01. (**c**) The stimulatory effect of DFO on HO-1 expression was demonstrated by immunofluorescence. Scale bars, 20 *μ*m

Since we have observed the anti-osteoclastogenesis function of DFO *in vivo* and *in vitro*, we next sought to explore the intrinsic mechanisms by which DFO mediated osteoclastogenesis. HO-1, the rate-limiting step in heme catabolism, which has been proved to be a negative regulator in osteoclastogenesis, so we hypothesized that DFO might regulate osteoclastogenesis by mediating HO-1 expression. To demonstrate our hypothesis, we first detected the effect of DFO on HO-1 expression. Osteoclastic precursor cells were treated with different concentrations of DFO in the presence of RANKL for 3 days, western blot and qRT-PCR analysis showed that DFO could induce HO-1 protein and mRNA expression in a dose-dependent manner (Figures 6a and b). Furthermore, the immunofluoresent analysis also demonstrated the stimulatory effect of DFO on HO-1 expression (Figure 6c). Taken together, all these results demonstrated DFO-induced HO-1 expression during osteoclastogenesis.

**Figure 7:**
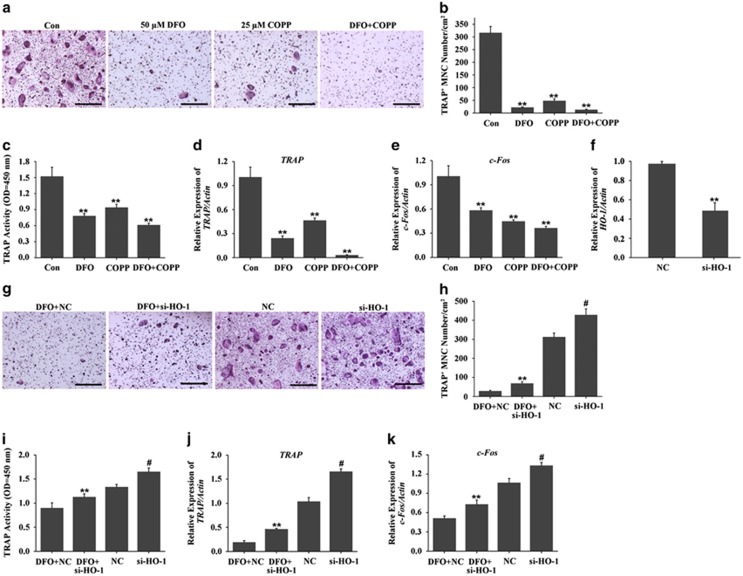
HO-1 was involved in DFO-inhibited osteoclastogenesis. (**a**) TRAP staining was performed to observe the effect of HO-1 activation on DFO-inhibited osteoclast formation. Scale bars, 50 *μ*m. (**b**) The number and area of TRAP-positive cells was counted. *n*=4, **P<0.01. (**c**) TRAP activity assessment was accomplished to observe the effect of HO-1 activation on DFO-inhibited osteoclast formation. *n*=4, **P<0.01. (**d**,**e**) qRT-PCR analysis of osteoclasts formation-specific genes *TRAP* and *c-Fos* in BMMs treated with DFO or/and COPP during osteoclastogenesis. *n*=4, **P<0.01. (**f**) Quantitative expression of HO-1 in the presence or absence of HO-1 siRNA treation. (**g**) TRAP staining was performed to observe depletion of HO-1 on DFO-inhibited osteoclast formation. (**h**) The number and area of TRAP-positive cells was counted. Scale bars, 50 *μ*m. *n*=4, **P<0.01*. # versus NC,* ^*#*^*P*<0.01. (**i**) TRAP activity assessment was accomplished to observe depletion of HO-1 on DFO-inhibited osteoclast formation. *n*=4, **P<0.01*. # versus NC,* ^*#*^*P*<0.01. (**j**–**k**) qRT-PCR analysis of osteoclasts formation-specific genes *TRAP* and *c-Fos* in BMMs treated with DFO or/and si-HO-1 during osteoclastogenesis. *n*=4, **P<0.01*. # versus NC,* ^*#*^*P*<0.01

Having observed DFO increased HO-1 expression, nextly we detected whether HO-1 was essential for DFO-inhibited osteoclastogenesis. Firstly, we performed gain-of-function experiment, in which we incubated osteoclast precursors with HO-1 inducer-cobaltprotoporphyrin (COPP). The TRAP staining and TRAP activity assay showed that activation of HO-1 by 25 *μ*M COPP significantly decreased osteoclasts formation. Furthermore, the inhibitory effect of DFO on osteoclastogenesis was also enhanced by COPP (Figures 7a–c). In addition, qRT-PCR analysis showed that the expression of *TRAP* and *c-Fos* were significantly decreased by COPP, which was further inhibited by DFO together with COPP (Figures 7d and e). Secondly, we performed loss-of-function experiment, in which we decreased the expression of HO-1 with si-HO-1. As evidenced by TRAP staining and TRAP activity assay, we found depletion of HO-1 could alleviate the inhibitory effect of DFO on osteoclasts formation, although the si-RNA against HO-1 did not completely reverse the effects of DFO (Figures 7f–i). Furthermore, inhibition of HO-1 could markedly attenuate DFO-decreased *TRAP* and *c-Fos* expression (Figures 7j and k). Taken together, all these results demonstrated that that HO-1 was an intermediator of DFO-inhibited osteoclastogenesis.

### DFO increased HO-1 expression by dampening p38MAPK pathway in osteoclast

**Figure 8:**
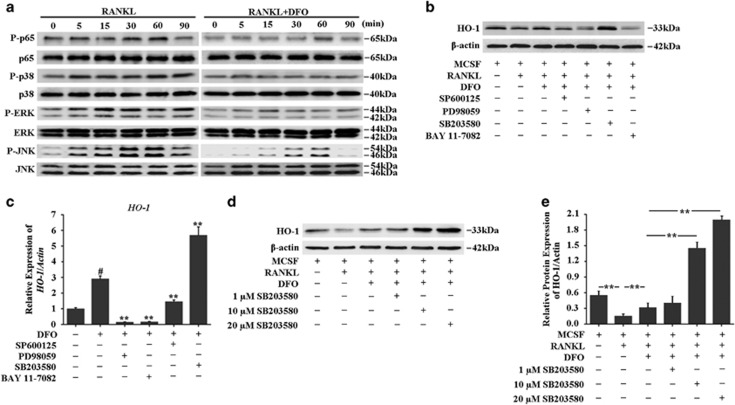
DFO increased HO-1 expression by dampening p38MAPK pathway. (**a**) Western blot analysis was performed to evaluate the effect of DFO on RANKL-induced the phosphorylation of p65, p38, ERK and JNK. (**b**) Western blot analysis was performed to evaluate the effect of blocking p38, JNK, NF-*κ*B and MEK on DFO-induced HO-1 expression. (**c**) qRT-PCR analysis was performed to evaluate the effect of blocking p38, JNK, NF-*κ*B and MEK on DFO-induced HO-1 expression. **P<0.01*. # versus* control, ^*#*^*P*<0.01. (**d**,**e**) Western blot analysis showed that SB203580 promoted DFO-induced HO-1 expression in a dose-dependent manner

Having identified HO-1 was required for DFO-inhibited osteoclastogenesis, we next sought to explore the molecular mechanisms involved in the induction of HO-1 by DFO. As it has been reported that mitogen-activated protein kinases (MAPKs) and nucleur factor-*κ*B (NF-*κ*B) are the key downstream pathways of RANKL in the process of osteoclastogenesis,^29, 30, 31^ we first explore the effects of DFO on RANKL-induced these intracellular signalings during the osteoclast differentiation. BMMs were preincubated with 50 *μ*M DFO, followed by stimulating with RANKL for the indicated time points. The results showed that phosphorylation of signal pathways, including p65, p38, ERK and JNK, were significantly activated by RANKL, whereas all of them were blocked by DFO in RANKL-stimulated osteoclasts (Figure 8a). To determine which signaling pathway was involved in DFO-induced HO-1 expression, we tested the effects of blocking these signaling pathways on DFO-induced HO-1 expression. The results showed that p38 inhibitor SB203580 significantly enhanced DFO-induced HO-1 expression. However, JNK inhibitor SP600125, NF-*κ*B inhibitor BAY 11-7082, and mitogen-activated protein/extracellular signal-regulated kinase (MEK) inhibitor PD98059 did not promote DFO-induced HO-1 expression (Figures 8b and c). Furthermore, we found that SB203580 promoted DFO-induced HO-1 expression in a dose-dependent manner (Figures 8d and e). Taken together, all these data suggested that inhibition of p38MAPK signaling pathway was involved in the induction of HO-1 by DFO.

## Discussion

Artificial joint replacement is widely used to treat severe joint degeneration. However, UHMWPE wear particles-induced osteolysis is a leading cause of early implant loosening in endoprosthetic surgery. Studies have showed that UHMWPE particles-induced osteolysis is due to enhanced osteoclasts differentiation and activity.^32^ Thus, therapeutic agents targeting osteoclasts formation are considered for treating wear particles-induced osteolysis. In the present study, we demonstrated for the first time that DFO, a powerful iron chelator, could significantly alleviate osteolysis in UHMWPE particles-induced mouse calvaria model by restraining inflammatory osteoclastogenesis. Furthermore, DFO was able to inhibit osteoclastogenesis in a dose-dependent manner. Mechanistically, DFO reduced osteoclasts formation by increasing HO-1 expression via p38MAPK signaling pathway. Taken together, we concluded that DFO might have great potential and value in treating wear particles-induced aseptic loosening.

With understanding of the pathogenesis of periprosthetic osteolysis, some effective preventative and nonsurgical interventions have been introduced. One large recent study indicates that early postoperative systemic administration of bisphosphonates can decrease the risk of aseptic loosening in total knee arthroplasty.^33, 34^ However, bisphosphonates have been proven unsuccessful in inflammatory conditions.^35^ Furthermore, it is reported that long-term administration of bisphosphonates could be associated with bone necrosis and atypical fractures in long bones.^36^ Therefore, the current appraisal of bisphosphonates to prevent loosening still needs for further study. Recently, TNF-*α* and IL-1 antagonists have variably been demonstrated efficacy in alleviating aseptic loosening, but come with unwanted immunosuppression.^35^ Denosumab (Amgen; Thousand Oaks, CA, USA), a monoclonal antibody against RANKL, has emerged as a potential therapeutic avenue for osteolysis, but the clinical trials show that it impacts immunocompetence less than originally thought.^35^ Thus, despite extensive research on drugs that target the inflammatory, osteoclastic and osteogenic responses to wear debris, it still needs for further studies to identify the more suitable treatment for wear particles-induced osteolysis.

DFO, an FDA-approved medication and a powerful iron chelator with 'hypoxia-mimetic' activity, was widely used as a therapeutic agent for treating iron overloaded-related diseases.^37^ Besides of exerting the anti-osteolysis function like bisphosphonates, IL-1 antagonists and Denosumab by inhibiting the process of osteoclastogenesis,^38^ DFO has been shown to increase angiogenesis via the hypoxia inducible factor (HIF) pathway. The HIF pathway activates angiogenesis as a regulator of response to hypoxia whose activation is also seen in skeletal repair.^39, 40^ In addition to promoting angiogenesis, DFO is also able to increase bone formation by enhancing osteoblasts activity.^24, 26^ Therefore, DFO has been emerged as a potential agent for treating bone regeneration and osteoporosis.^41, 42^ In this study, mouse calvaria osteolysis model was used to examine the effect of DFO on particles-induced aseptic loosening *in vivo*. Both micro-CT and histological assessments demonstrated that DFO significantly protected from UHMWPE particles-induced osteolysis. Meanwhile, DFO treatment could alleviate particles-induced bone destruction and osteolysis, which were confirmed to associate with particles-promoted osteoclastogenesis. Our results for the first time demonstrated that DFO could be effectively used for the treatment of wear particles-induced osteolysis *in vivo*. Thus, DFO might be used as a therapeutic agent for treating wear particles-induced aseptic loosening.

In the present study, we confirmed that DFO obviously inhibited osteoclasts formation at early stage (days 0–3), whereas adding DFO to osteoclastic precursor cells at late stage did not affect osteoclasts formation. Indeed, Leger *et al.*^43^ also found that DFO was not shown to decrease osteoclasts numbers, which might be caused by adding DFO for the last day of the human osteoclast assays. Furthermore, Philipp *et al.*^44^ added DFO in the beginning of the osteoclastogenesis assay with cells of rodent origin, resulting in a significant suppression of osteoclasts differentiation. Consistent with these findings, our studies further demonstrated that the inhibition of osteoclast formation by DFO was due to dampen osteoclast progenitor cells differentiation.

RANKL-induced osteoclast differentiation is associated with the upregulation of specific genes, including *TRAP*, *c-Fos*, *Cathepsin K*, *DC-STAMP*, *V-ATPase a3* and *V-ATPase d2.*^28^ Data from this study showed that these RANKL-induced specific genes expression were obviously attenuated by DFO in a time-dependent manner. Of note, *c-Fos* as a critical transcript factor for osteoclastogenesis was markedly increased at early stage (day 1), whereas the induction of *c-Fos* expression by RANKL was alleviated from days 1 to 5, indicated that *c-Fos* might be an early marker gene for osteoclast formation. Indeed, c-Fos as a major regulator of osteoclastogenesis conducts the expression of osteoclast specific genes, such as *TRAP, Cathepsin K*, *DC-STAMP*, *V-ATPase a3* and *V-ATPase d2*. In the current study, inhibition of these specific genes expressions by DFO further provided evidence of DFO-inhibited osteoclast formation.

Previous studies suggest that overproduction or inadequate removal of ROS may be involved in the formation of fibrotic pseudocapsular tissues around revised total hip replacement components,^45^ suggesting that ROS-induced oxidative stress has an important role in wear particles-induced osteolysis. HO-1, as an inducible enzyme, which is involved in oxidative stress processes. In bone tissue, HO-1 mRNA is expressed in osteoblasts, osteocytes and osteoclasts.^46^ Several studies have elucidated the role of HO-1 in osteoclastogenesis. Ke *et al.*^46^ found that HO-1-deficiency synergized with RANKL signaling to increase the number and activity of osteoclasts. Induction of HO-1 could inhibit osteoclast differentiation via MAP kinase.^19^ Furthermore, Eiko *et al.*^47^ demonstrated that RANKL induced osteoclasts differentiation by inhibiting HO-1 expression via activation of p38 MAPK signaling pathway. In the present study, we confirmed for the first time that HO-1 was involved in DFO-inhibited osteoclasts formation.

Even though many studies have confirmed that DFO could inhibit osteoclast differentiation and activity.^24^ However, little is known regarding how DFO regulates osteoclastogenesis. In the process of osteoclast differentiation, RANKL binding to its receptor RANK leads to the activation of downstream signaling molecules, such as MAPKs (ERK1/2, p38 and JNK1/2) and NF-*κ*B.^48, 49^ Previous studies have showed that the formation of osteoclasts can be reduced by inhibition of JNK, ERK and p38, suggesting these molecules are critical for RANKL-induced osteoclastogenesis.^50^ Furthermore, RANKL stimulation triggers the induction of the NF-*κ*B heterodimer p65 (RelA)/p50 (NF-*κ*B1), which induces the expression of NFATc1, a transcription factor that regulates the terminal RANKL-induced differentiation of osteoclasts.^51^ In our study, we found that DFO could downregulate ERK, JNK, p38 and p65 activation in osteoclast differentiation, evidenced by little ERK, JNK, p38 and p65 phosphorylation after DFO treatment. Further studies found that inhibition of p38 signaling pathway could promote DFO-induced HO-1 expression, indicating that p38 was involved in DFO-induced HO-1 expression. Our study delineated a previously unknown mechanism that DFO inhibited UHMWPE particles-induced osteolysis by restraining inflammatory osteoclastogenesis through upregulation of HO-1 via p38MAPK pathway. However, as the results showed in Figure 7, HO-1 depletion by siRNA did not completely reverse the effects of DFO, which revealed that DFO restrained the inflammatory osteoclastogenesis might through the other alternative pathway. As studies have revealed that MAPKs (including p38MAPK, JNK and ERK) and NF-*κ*B are critical for RANKL-induced osteoclastogenesis,^50^ and our results in Figure 7 have demonstrated that DFO significantly dampens the activation of MAPKs and NF-*κ*B induced by RANKL. In addition, it has been reported that clinoquinol, another iron chelator, impairs RANKL-driven AKT phosphorylation and NFATC1 activation in the process of osteoclastogenesis,^38^ both AKT and NFATC1 are required for efficient osteoclastogenesis and osteoclast activation.^52, 53, 54^ Except of p38MAPK, we predict that DFO inhibits osteoclastogenesis may also by regulating RANKL-induced ERK, JNK, AKT or NFATC1 activation.

The mouse calvaria osteolysis model is widely used to explore the mechanisms of UHMWPE particles-induced osteolysis. However, some deficiencies exist in this model. First, mechanical loading may affect UHMWPE particles-induced osteolysis in patients with endoprosthetic surgery, whereas which is not considered in the mouse calvaria osteolysis. Second, the size of UHMWPE particles used to generate mouse model was uniform, whereas UHMWPE particles from artificial joint are not identical. Thus, future studies are needed to further explore the most suitable mouse model for UHMWPE particles-induced osteolysis.

In conclusion, in the present study, we demonstrated that UHMWPE particles-induced osteolysis could be alleviated by DFO via restraining of inflammatory osteoclasts formation and activity. Furthermore, the inhibitory effects of DFO on osteoclastogenesis, which were achieved mainly through induction of HO-1 expression. Further study confirmed that DFO induced HO-1 expression via inhibition of p38 signaling pathway, resulting in the reduction of osteoclasts formation. Taken together, we concluded that DFO might be used as an innovative and safe therapeutic alternative for treating wear particles-induced aseptic loosening.

## Materials and Methods

### Materials

Alpha modification of Eagle medium (*α*-MEM), penicillin/streptomycin and fetal bovine serum (FBS) were purchased from Gibco-BRL (Sydney, NSW, Australia). Recombinant soluble mouse M-CSF (Catalog#315-02) and mouse RANKL (Catalog#315-11) were purchased from Peprotech (Rocky Hill, USA). The cell counting kit (CCK-8) was obtained from Do jin do Molecular Technology (Kumamoto, Japan). Specific antibodies against *β*-actin, extracellular signal regulated kinase (ERK), phospho-ERK (Thr202/Tyr204), c-Jun N-terminal kinase (JNK), phospho-JNK(Thr183/Tyr185), p38, phospho-p38 (Thr180/Tyr182), NF-*κ*B p65, phospho-NF-*κ*B p65 (Ser536) were purchased from Cell Signaling Technology (Cambridge, MA, USA). HMOX1 (HO-1) polyclonal antibody (Catalog#10701-1-AP) was provided by Proteintech (Rosemont, USA). PD98059, SB203580, SP600125, BAY11-7082 were purchased from Selleck Chemicals (USA). Cobalt protoporphyrin (COPP) IX was purchased from Frontier Scientific (Logan, UT, USA). Desferrioxamine (DFO), the tartrate-resistant acid phosphatase (TRAP) staining kit and all other reagents were purchased from Sigma Aldrich (St Louis, MO, USA) unless stated otherwise.

### Methods

#### Preparation of UHMWPE particles

UHMWPE particles were provided by the manufacturer (Zimmer Inc., Warsaw, IN, USA). The characteristics of the particle's morphology have been published previously.^55^ The mean diameter of these particles was 2.6 *μ*m (range from <0.7 to 21 *μ*m). To avoid contamination with endotoxins, the particles were washed three times with 70% ethanol and sterilized for 72 h to remove endotoxin and heat sterilized, then dispersed in PBS at 2 × 10^8^ particles per ml. Endotoxin levels of the particle suspension were determined by a Limulus assay according to the manufacturer's instructions.

#### UHMWPE-induced calvarial osteolysis model

A wear particle-induced mouse calvarial osteolysis model was generated as previously described.^1^ Animal studies were performed in accordance with the principles and procedures approved by the Animal Care Committee of Shanghai Jiao Tong University. Briefly, 24 healthy male 8-week-old C57BL/6J mice were randomly divided into to four groups: sham PBS control (Sham), UHMWPE particles with PBS (Vehicle), and UHMWPE particles with 10 mg/kg (low) and 30 mg/kg (high) concentrations of DFO. The mice were anesthetized, and the cranial periosteum was separated from the calvarium by sharp dissection. Then, 100 ul of particle suspension was uniformly spread over the periosteum at the middle suture of the calvaria in vehicle, low and high group, whereas sham group not. Two days after implantation of UHMWPE particles, PBS or DFO was injected every day intraperitoneally, respectively for 14 days. The animals were housed 5 per cage and were maintained under a strict 12 h light: 12 h darkness cycle at 22 °C with standard mice food pellets and had free access to tap water. At the end of the experiment, the mice were sacrificed, and the calvaria were excised and fixed in 4% paraformaldehyde for micro-computed tomography (CT) and histological analysis. No adverse events were found during the generation of mouse calvarial osteolysis model.

#### Bone resorption assay and F-actin ring formation assay

The bone resorption assay was conducted as previously described.^28^ Briefly, BMM cells were plated onto bovine bone slices in 96-well plates at a density of 1 × 10^4^cells/well. The BMM cells were cultured with complete *α*-MEM medium supplemented with M-CSF (30 ng/ml), RANKL (50 ng/ml) and different concentrations of DFO. Cell culture media were replaced every 2 days until mature osteoclasts had formed. On 10 days, the osteoclasts were removed from the bone slices by mechanical agitation and sonication. Resorption pits stained with toluidine blue were photographed under a high-quality microscope. Three view fields were randomly selected for each bone slice for further analysis. The percentage of resorbed bone surface area was counted using the Image J software. Experiments were repeated independently at least three times.

To perform F-actin ring formation assay, osteoclasts treated with various concentrations of DFO were fixed with 4% paraformaldehyde for 15 min, permeabilized for 5 min with 0.1% Triton X-100, and incubated with rhodamine-conjugated phalloidin (Invitrogen Life Technologies, Grand Island, NY, USA) for 30 min at room temperature and then washed extensively with PBS three times. The F-actin ring distribution was visualized using a fluorescence microscope (ZEISS, Jena, Germany), and the average number of F-actin ring was calculated.

#### Organ culture and cytokines detection

The murine calvarias culture is according to the report before.^56^ The dissected calvarial tissue samples were weighted and cultured in serumless medium (10 ml/g weight) (Dulbecco's Modified Eagles Media, Life Technologies, Gaithersburg, MD, USA) containing 1% Penicillin/Streptomycin for 72 h at 37 °C with 5% CO2. The release of IL-1*β*, IL-6 and TNF-*α* from dissected murine calvaria into the medium was measured with the enzyme-linked immunoassay (ELISA) kit specific for mice IL-1b; IL-6 and TNF-a (Duoset R&D Systems, Abingdon, UK).

#### Micro-CT imaging analysis

The fixed calvarias were analyzed using a high-resolution micro-CT scanner (Skyscan 1172; Skyscan; Aartselaar, Belgium). All calvarias were scanned according to the same parameters (pixel size, 9 *μ*m; X-ray voltage, 50 kV; electric current, 500 *μ*A; rotation step, 0.7°). After reconstruction, a spherical volume of interest (VOI) of 3 mm in diameter around the midline suture was selected for further qualitative and quantitative analysis. Bone mineral density (BMD), bone volume against tissue volume (BV/TV) and total volume of pore space of each sample were measured.

#### Histological analysis

After micro-CT scanning, the samples were decalcified in 10% EDTA for 3 weeks and then dehydrated, embedded in paraffin. Histological sections (5 *μ*m thick) were prepared for H&E and TRAP staining. The specimens were then examined and photographed under a high-quality microscope. The numbers of TRAP-positive multinucleated osteoclasts were counted in each sample.

#### Cell viability assay

The cytotoxic effects of DFO on BMMs viability were determined using a CCK-8 assay according to the manufacturer's instructions. The BMM cells were plated in 96-well plates at a density of 5 × 10^3^ cells/well, and cultured in complete *α*-MEM medium supplemented with 30 ng/ml M-CSF, and treated with different concentrations of DFO (0, 6.25, 12.5, 25, 50, 100, 200 and 400 *μ*M) for 48 h. Next, changed the medium of each well with 10 *μ*l CCK-8 and 100 *μ*l *α*-MEM medium, then incubated at 37 °C for an additional 1.5 h. The optical density (OD) was then measured at a wavelength of 450 nm with an ELX680 absorbance microplate reader (Bio-Tek, Winooski, USA).

#### Bone marrow-derived macrophage isolation and osteoclast culture

Primary BMMs were isolated from the long bones of 8-week-old C57BL/6J mice. cells were isolated from the femur and tibiae bone marrow and cultured in a 100mm dish with complete *α*-MEM medium in the presences of 10 ng/ml M-CSF for 24 h. Non-adherent cells were harvested and cultured with fresh medium containing 50 ng/ml M-CSF. Three days later, the adherent cells were harvested as osteoclasts precursors (pre-osteoclasts). These cells were then seeded and further cultured with complete *α*-MEM medium containing M-CSF (30 ng/ml) and RANKL (50 ng/ml) for 3–5 days with various concentrations of DFO (0, 12, 25, 50 *μ*M). Cell culture media were replaced every two days until mature osteoclasts had formed. Next, cells were washed twice by PBS and fixed with 4% paraformaldehyde for 15 min and then stained for TRAP activity. TRAP-positive cells with three or more nuclei were counted under a microscope.

#### Immunofluorescence staining

BMM cells were seeded onto the sterile cover slips at a density of 5 × 10^4^ cells/well in 24-well plates, and cultured with complete *α*-MEM medium supplemented with M-CSF (30 ng/ml), RANKL (50 ng/ml), and 50 *μ*M DFO. After incubation, cells were fixed in 4% paraformaldehyde for 10 min, treated with 0.1% Triton X-100 for 15 min and then incubated in 3% bovine serum albumin (BSA)/ PBS for 30 min at room temperature. Next, cells were incubated with mouse anti-HO-1 antibody (1:100 dilution) at 4 °C overnight. Cell nuclei were counterstained with Hoechst 33258 at room temperature for 15 min in the dark. Images were acquired using a fluorescence microscope (ZEISS Axio Imager A2, Carl Zeiss microscopy GmbH).

#### RNA interference

The small interfering RNA (siRNA) oligonucleotide for HO-1 was designed and synthesized by GenePharma (Shanghai, China). The targeting sequences of murine HO-1 siRNA (si-HO-1) were as follows: forward 5′-CCACACAGCACUAUGUAAATT-3′ and reverse 5′-UUUACAUAGUGCUGUGUGGTT-3′. BMM cells cultured with or without DFO in the presence of RANKL in antibiotic-free media were transfected with 100 nM si-HO-1 using lipofectamine 3000 (Invitrogen) according to the manufacturer's instructions. The sequences of negative control (NC) were as follows: forward 5′-UUCUCCGAACGUGUCACGUTT-3′ and reverse 5′-ACGUGACACGUUCGGAGAATT-3′. After incubation for 48 h, the cells were harvested to extract total RNA for RT-PCR. For TRAP staining, we incubated the cells for another 5 days.

#### RNA extraction and quantitative real-time PCR (qRT-PCR)

To measure specific gene expression during osteoclast formation, we performed quantitative PCR assay. Briefly, cells were seeded in six-well plates at a density of 1 × 10^5^ cells per well and cultured in complete *α*-MEM medium supplemented with 30 ng/ml M-CSF and 50 ng/ml RANKL. After treatments with various concentrations of DFO, COPP or siRNA, total RNA was isolated from BMM cells using Trizol reagent (Invitrogen) according to the manufacturer's instruction. Next, cDNA was synthesized from 1 *μ*g of total RNA using reverse transcriptase (TakaRa, Shiga, Japan). qRT-PCR was performed to amplify the cDNA using the SYBR Premix Ex Tag kit (TaKaRa) and an ABI 7500 Sequencing Detection System (Applied Biosystems, Foster City, CA, USA). The following cycling conditions were used: 40 cycles of denaturation at 95 °C for 5 s and amplification at 60 °C for 24 s. *β*-actin was used as the house keeping gene, and all reactions were run in triplicate. The mouse primer sequences for *TRAP* (*Accession Numbers: NM_011611*), *c-Fos* (*Accession Numbers: NM_010234*), *Cathepsin K* (*Accession Numbers: NM_ 007802*), *DC-STAMP* (*Accession Numbers: NM_001289513*), *V-ATPase α3* (*Accession Numbers: NM_016921*), *V-ATPase d2* (*Accession Numbers: NM_175406*), *HO-1* (*Accession Numbers: NM_010442*) and *β-actin* (*Accession Numbers: NM_007393*) were described in Supplementary Table 1.

#### Western blot analysis

BMM cells were seeded in six-well plates at a density of 1 × 10^5^ cells per well. After various treatments in the presence of M-CSF and RANKL, cells were washed with PBS and lysed in ice-cold lysis buffer (Cell Signaling Technology) supplemented with cocktail for 30 min. Next, the lysates were centrifuged at 12 000 × *g* for 15 min, and the supernatants that contained the proteins were harvested. Protein concentrations were determined by a BCA protein assay kit (Pierce Biotechnology, Rockford, IL, USA). Equal amounts of protein lysates were resolved using SDS-PAGE on 10% gels, and transferred to PVDF membranes (Millipore, Bedford, MA, USA). Afterwards, the membranes were blocked with 5% skimmed milk solution for 1 h, and then incubated with primary antibodies diluted in 1% BSA powder in TBS-Tween (TBST) overnight at 4 °C. The membranes were then washed three times with TBST solution and incubated with the appropriate secondary antibodies. The antibody reactivity was visualized using the enhanced chemiluminescence detection system as recommended by the manufacturer. Signal intensities were quantified using Image-J software (Bethesda, MD, USA).

### Statistical analysis

Data were collected from three or more independent experiments and expressed as mean±S.D. A two-sided Student's *t*-test was used to analyze the difference between groups. One-way analysis of variance was performed to show the difference between groups. *P*<0.05 was considered significantly different.

## Abbreviations

DFO: Desferrioxamine
UHMWPE: Ultrahigh-molecular-weight polyethylene
HO-1: Heme oxygenase-1
COPP: Cobalt protoporphyrin
MAPKs: Mitogen-activated protein kinases
RANKL: Receptor activator of nuclear factor-*κ* B ligand
RANK: Receptor activator of nuclear factor-*κ* B
M-CSF: Macrophage-colony stimulating factor
IL-1: Interleukin-1
IL-6: Interleukin-6
PGE2: Prostaglandin E2
TNF-*α*: Tumor necrosis factor-*α*
NFATc1: Nuclear factor of activated T-cells 1
ROS: Reactive oxygen species
ROI: Region of interest
BMMs: Bone marrow derived macrophages
TRAP: Tartrate-resistant acidic phosphatase
CCK-8: Cell Counting Kit-8
*μ*M: Micromole
DC-STAMP: Dendritic cell-specific transmembrane protein
qRT-PCR: Quantitative real-time polymerase chain reaction
NF-*κ*B: Nucleur factor-*κ*B
ERK: Extracellular signalregulated kinase
JNK: c-Jun N-terminal kinase
MEK: Mitogen-activated protein/extracellular signal-regulated kinase
FDA: Food and Drug Administration
HIF: Hypoxia inducible factor
*α*-MEM: *α*-modification of Eagle medium
FBS: Fetal bovine serum
VOI: Volume of interest
BMD: Bone mineral density
BV/TV: Bone volume against tissue volume
H&E: Hematoxylin and Eosin
OD: Optical density
TBST: Tris buffer saline Tween
